# Comparison of the Usefulness of Covered and Uncovered Laser‐cut Metal Stents

**DOI:** 10.1002/deo2.70208

**Published:** 2025-10-01

**Authors:** Toshio Fujisawa, Masao Toki, Kei Saito, Yuta Hasegawa, Eisuke Iwasaki, Michihiro Saito, Katsuya Kitamura, Ryosuke Tonozuka, Takao Itoi, Ken Ito, Keiko Kaneko, Naminatsu Takahara, Tadakazu Hisamatsu, Hiroyuki Isayama

**Affiliations:** ^1^ Department of Gastroenterology Graduate School of Medicine Juntendo University Bunkyo Japan; ^2^ Department of Gastroenterology and Hepatology Kyorin University School of Medicine Mitaka Japan; ^3^ Department of Gastroenterology Graduate School of Medicine, The University of Tokyo Bunkyo Japan; ^4^ Department of Gastroenterology Nippon Medical School Tokyo Japan; ^5^ Department of Internal Medicine Division of Gastroenterology and Hepatology Keio University School of Medicine Tokyo Japan; ^6^ Department of Internal Medicine, Division of Gastroenterology and Hepatology Toho University Ohashi Medical Center Tokyo Japan Tokyo Japan; ^7^ Department of Gastroenterology and Hepatology Tokyo Medical University Hachioji Medical Center Tokyo Japan; ^8^ Department of Medicine Division of Gastroenterology Showa Medical University of Medicine Tokyo Japan; ^9^ Department of Gastroenterology and Hepatology Tokyo Medical University Tokyo Japan

**Keywords:** covered, laser‐cut metal stent, malignant distal biliary obstruction, removability, uncovered

## Abstract

**Objectives:**

This study aimed to evaluate the clinical characteristics of laser‐cut self‐expandable metal stents (SEMS) and to compare the outcomes between covered and uncovered laser‐cut SEMS for malignant distal biliary obstruction (MDBO).

**Methods:**

A multicenter retrospective analysis was conducted across eight Japanese university hospitals, including 124 patients (81 with covered and 43 with uncovered SEMS). Treatment outcomes, recurrent biliary obstruction (RBO), survival, and treatment‐related adverse events (tAEs) were compared.

**Results:**

The rates of technical success (100% vs. 100%) and clinical success (94% vs. 98%) were comparable between the two groups. However, the incidence of RBO was significantly higher in the uncovered SEMS group compared to the covered SEMS group (37% vs. 20%, *p* = 0.034), particularly due to stent occlusion (37% vs. 14%, *p* = 0.005). Nevertheless, there were no significant differences in time to RBO (567 days vs. 459 days) or overall patient survival (277 days vs. 227 days) between the groups. The overall rate of tAEs was similar (15% vs. 12%), though pancreatitis was observed exclusively in the covered SEMS group, with a trend toward lower incidence in the uncovered group (10% vs. 0%, *p* = 0.050). Stent removal was performed only in patients with covered SEMS, and all 13 removal attempts were successful.

**Conclusion:**

Laser‐cut SEMS demonstrated similar efficacy to braided stents in the management of MDBO. The covered laser‐cut SEMS was associated with a lower RBO rate than the uncovered SEMS. Additionally, Laser‐cut SEMS can be removed if it is fully covered.

## 1 Introduction

Self‐expandable metal stents (SEMS) are effective in relieving obstructive jaundice caused by malignant distal biliary obstruction (MDBO) and are typically placed transduodenally [1, 2, 3]. Biliary metal stents are broadly classified into two types based on their construction: laser‐cut SEMS, which are carved from a metal tube using a laser, and braided stents, which are woven from multiple wires [4]. While braided SEMS are more commonly available and widely used, laser‐cut SEMS offer potential advantages in certain cases [5]. Recently, laser‐cut SEMS have been recharacterized and are increasingly employed in procedures such as endoscopic ultrasound‐guided biliary drainage.

First, laser‐cut stents have a thinner delivery system, facilitating endoscopic handling and allowing easier passage through strictures [6]. Second, they exhibit minimal shortening upon deployment, potentially reducing placement failures, especially among less‐experienced endoscopists [7]. Additionally, laser‐cut stents have multiple sharp points along the stent body, which may help prevent migration, even in covered stents [8, 9, 10]. However, a major concern is the difficulty of removal if stent occlusion occurs [8, 11].

Despite these advantages and concerns, few studies have comprehensively examined the clinical characteristics of laser‐cut stents, and the differences between covered and uncovered laser‐cut stents remain unclear [12, 13, 14]. Therefore, this study aims to analyze the clinical performance of laser‐cut stents in MDBO treatment and to compare the outcomes of covered and uncovered laser‐cut stents using a single stent model with an identical structure.

## 2 Methods

### 2.1 Study Design

This multicenter retrospective study analyzed laser‐cut metal stent placement for MDBO at eight Japanese university hospitals. The study was approved by the ethics committee at each participating institution (H20‐0002). The study protocol was publicly accessible for opt‐out on the participating institutions’ websites.

### 2.2 Patient Selection

#### 2.2.1 Inclusion Criteria

This study included consecutive patients who underwent covered or uncovered laser‐cut metal stent (X‐SUIT NIR; Olympus Corporation, Tokyo, Japan) placement for unresectable MDBO between September 2014 and December 2018. All patients had MDBO confirmed by endoscopic retrograde cholangiopancreatography (ERCP), magnetic resonance cholangiopancreatography (MRCP), or computed tomography (CT) and presented with obstructive jaundice or cholangitis requiring biliary drainage. Malignancies were confirmed through pathological examination.

#### 2.2.2 Exclusion Criteria

Patients younger than 20 years, pregnant individuals, and those with stents placed outside the bile duct were excluded.

### 2.3 Procedure

Endoscopic stent placement was performed under conscious sedation with midazolam and pethidine hydrochloride. A duodenoscope (TJF‐260 V, JF‐260 V [Olympus Medical Systems, Tokyo, Japan], or ED‐580T [Fujifilm Medical Corp., Tokyo, Japan]) was used for the procedure.

Endoscopic retrograde cholangiopancreatography (ERCP) was primarily conducted with an MTW catheter (MTW Endoskopie, Wesel, Germany) or a Swish catheter (Swish, TSK Laboratory, Japan). Guidewires, including the 0.025‐inch VisiGlide 2 (Olympus Medical Systems), EndoSelector (Boston Scientific, Marlborough, MA, USA), and 0.035‐inch Seekmaster (Piolax, Tokyo, Japan), were used for bile duct identification and SEMS insertion.

Endoscopic sphincterotomy (EST), endoscopic papillary balloon dilation (EPBD), or endoscopic sphincterotomy followed by balloon dilation (ESBD) [15, 16, 17] was performed when necessary using the CleverCut3 (Olympus Corporation) or Hurricane (Boston Scientific Corporation, Boston, USA).

Tumor invasion of the cystic duct orifice was defined based on tumor extension around the cystic duct as confirmed by CT or MRCP, or the presence of biliary irregularities causing cystic duct narrowing as observed by ERCP.

**FIGURE 1:**
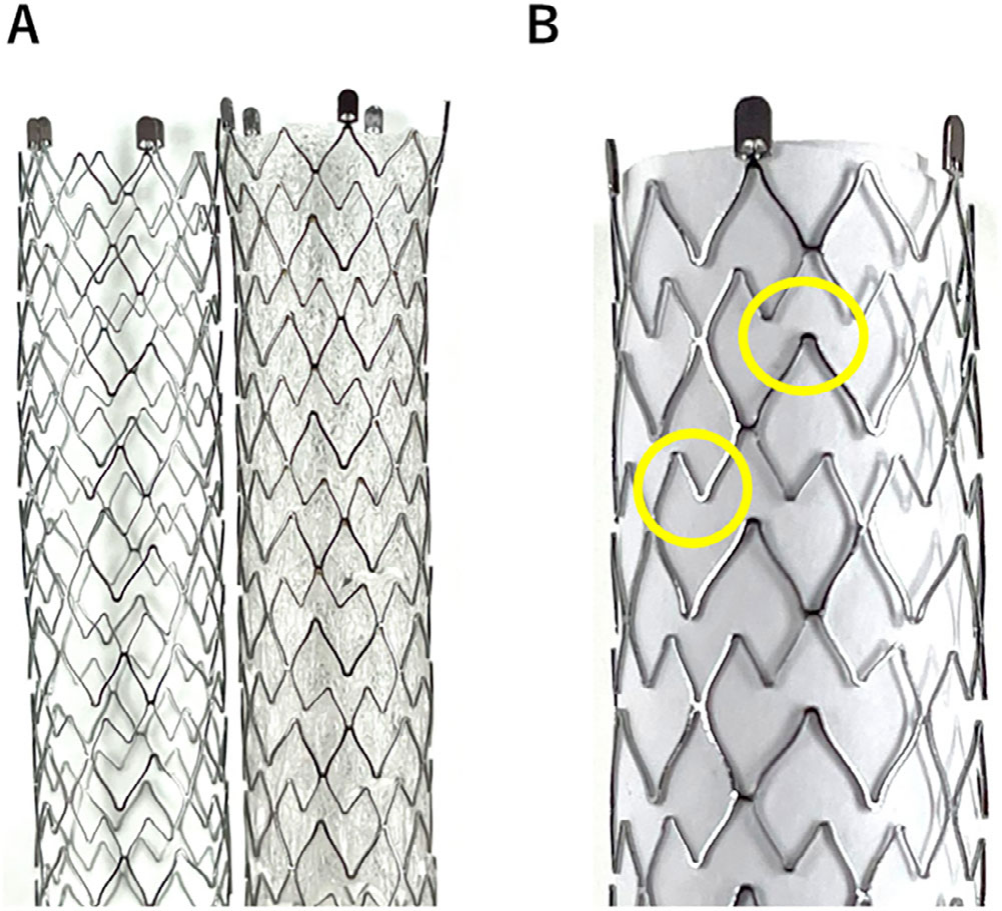
Images of the laser‐cut self‐expandable metal stent (SEMS), X‐SUIT NIR, used in the present study. (A) Images of covered (left) and uncovered (right) X‐SUIT NIR. Both stents have the same structural design, with the covered stent featuring a silicone and polyurethane membrane on its outer surface. (B) High‐magnification view of a segment of the uncovered laser‐cut SEMS. Yellow circles indicate pointed shapes.

In this study, we aimed to compare the clinical efficacy of covered and uncovered laser‐cut self‐expandable metal stents (SEMS) using the X‐SUIT NIR, which has an identical structure except for the presence of a covering. Both covered and uncovered X‐SUIT NIR stents are fabricated by laser‐cutting a nickel‐titanium alloy. The covered X‐SUIT NIR features a silicone and polyurethane membrane on its outer surface (Figure 1). The stent was deployed to cover the biliary stricture, with its distal end positioned either across or above the duodenal papilla. Re‐intervention was performed in cases of recurrent biliary obstruction (RBO) due to stent‐related complications or when stent removal was required due to adverse events (AEs). The decision to remove the existing laser‐cut metal stent during re‐intervention was left to the discretion of the operator. All procedures were performed or supervised by experts with experience in more than 1000 ERCP cases.

### 2.4 Definitions and Outcomes

AEs and outcome definitions were based on the Tokyo Criteria 2024 [18]. The primary outcome was RBO, including both the incidence rate and the time to RBO (TRBO). Secondary outcomes included the removability of the laser‐cut metal stent, technical success rate, clinical success rate, and AE rate as defined by the Tokyo Criteria 2024 [18].

Technical success was defined as the successful placement of the stent in the intended location. Clinical success was defined as a 50% reduction or normalization of bilirubin levels or the resolution of cholangitis within 14 days after stent placement.

AEs were defined as any events requiring conservative treatment, medication, intervention, or hospitalization, in addition to RBO. The total number of each AE was recorded, and the overall AE rate was calculated as the number of patients experiencing at least one AE divided by the total number of patients.

RBO was defined as a composite endpoint, including stent occlusion or migration resulting in recurrent biliary obstruction or other conditions requiring biliary drainage or stent removal. TRBO was estimated using Kaplan‐Meier curves and compared between the two groups using the log‐rank test [13]. Stent migration was defined as occurring at the time when migration‐related symptoms appeared; asymptomatic stent migration cases were not treated as censored in the TRBO analysis. Death without RBO and non‐RBO AEs requiring stent removal were considered censored cases at the time of death and stent removal, respectively.

### 2.5 Statistical Analysis

Clinical data were collected until May 31, 2020, and subsequently finalized. Due to the revision of the Clinical Research Act in 2021, ethical considerations in the study design were modified during the study period. As a result, some ethics committees required additional time to reapprove the research.

Statistical analysis was performed using SPSS software version 30.0 (IBM Corp., Armonk, NY, USA). Categorical variables were analyzed using the chi‐square test or Fisher's exact test. Continuous variables are presented as medians with interquartile ranges (IQRs) and were analyzed using the Mann‐Whitney U test. Factors associated with RBO that showed significance in the univariate analysis were further evaluated using binary logistic regression. A *p*‐value of < 0.05 was considered statistically significant.

## 3 Results

### 3.1 Patient Characteristics

A total of 124 patients were enrolled in this study (Table 1). The cohort included 77 males (62%) and 47 females (38%). The median patient age was 73 years (interquartile range [IQR]: 66–81).

**TABLE 1:** Patient background.

Factors		All (Covered + Uncovered) (n = 124)	Covered (n = 81)	Uncovered (n = 43)	Covered vs Uncovered *p*‐value
Sex	Male/Female	77 (62%)/47 (38%)	48 (59%)	29 (67%)	0.371
Age^*^		73 (66 ‐ 81)	73 (67 ‐ 81)	71 (65 ‐ 82)	0.597
Naïve papilla		89 (72%)	51 (63%)	38 (88%)	< 0.001
Prior biliary drainage		36 (29%)	30 (37%)	6 (14%)	< 0.001
Primary cancer					0.109
	Pancreas	68 (55%)	46 (57%)	22 (51%)	—
	Bile duct	25 (20%)	13 (16%)	12 (28%)	—
	Gallbladder	6 (5%)	2 (2%)	4 (9%)	—
	Gastric	5 (4%)	5 (6%)	0	—
	Esophagus	5 (4%)	5 (6%)	0	—
	Duodenal papilla	4 (3%)	2 (2%)	2 (5%)	—
	Lung	4 (3%)	2 (2%)	2 (5%)	—
	Colon	4 (3%)	4 (5%)	0	—
	Others	3 (3%)	2 (2%)	1 (2%)	—
Length of stricture ^*^		20 (15 ‐ 35)	19 (15 ‐ 30)	27 (16 ‐41)	0.017
Dilation of the pancreatic duct		67 (54%)	45 (56%)	22 (51%)	0.640
Invasion to the cystic duct		45 (36%)	29 (36%)	16 (37%)	0.916
Invasion to the duodenum		26 (21%)	18 (22%)	8 (19%)	0.638
Chemotherapy		69 (56%)	42 (52%)	27 (63%)	0.243
Antithrombotic drugs		24 (19%)	14 (17%)	10 (23%)	0.423
	Antiplatelet drugs	14 (11%)	8 (10%)	6 (14%)	—
	Anticoagulant drugs	10 (8%)	6 (7%)	4 (9%)	—

* median (interquartile range)

Eighty‐nine patients (72%) had never undergone ERCP and were classified as having a naïve papilla. In contrast to naïve papilla cases, in almost all non‐naïve papilla cases (35 patients), biliary drainage had been performed with either a plastic stent or a naso‐biliary tube before laser‐cut stent placement. The frequency of prior drainage was significantly higher in the covered stent group, while in the uncovered stent group, only one case underwent prior drainage via the percutaneous transhepatic route. The primary etiologies of malignant distal biliary obstruction were pancreatic cancer in 68 patients (55%), bile duct cancer in 25 (20%), gallbladder cancer in six (5%), and other types of cancer in 25 (20%).

The median length of biliary stricture was 20 mm (IQR: 15–35), and 67 patients (54%) exhibited pancreatic duct dilation. Tumor invasion of the cystic duct orifice was observed in 45 patients (36%), while duodenal invasion was present in 26 patients (21%).

Sixty‐nine patients (56%) received chemotherapy, and 24 (19%) were on antithrombotic therapy.

### 3.2 Results of Stent Treatment

Technical success was achieved in all patients (100%), and clinical success, defined as improvement in jaundice, was achieved in 118 patients (95%) (Table 2).

**TABLE 2:** Results of the treatment.

Factors		All (Covered + Uncovered) (*n* = 124)	Covered (*n* = 81)	Uncovered (*n* = 43)	Covered vs Uncovered *p*‐value
Technical success		124 (100%)	81 (100%)	43 (100%)	1.000
Clinical success		118 (95%)	76 (94%)	42 (98%)	0.664
Scope type					
	Duodenal / Balloon‐assisted	118 (95%)/ 6 (5%)	75 (93%)/ 6 (7%)	43 (100%)/ 0	0.092
Stent types					
	Length 4/ 6/ 8/ 10 cm	2 (2%)/ 43 (34%)/ 77 (62%)/ 2 (2%)	1 (1%)/ 29 (36%)/ 51 (63%)/ 0	1 (2%)/ 14 (33%)/ 26 (60%)/ 2 (5%)	0.252
	Diameter 8/ 10 mm	18 (15%)/ 106 (85%)	5 (6%)/ 76 (94%)	13 (30%)/ 30 (70%)	< 0.001
Manipulation of papilla					
	EST/EPBD/ ESBD/ None	74 (60%)/ 5 (4%)/ 23 (18%)/ 22 (18%)	38 (47%)/ 3 (4%)/ 23 (28%)/ 17 (21%)	36 (84%)/ 2 (5%)/ 0 (0)/ 5 (11%)	< 0.001
Method of stent placement					
	Across/Above the papilla	85 (69%)/ 39 (31%)	53 (65%)/ 28 (35%)	32 (74%)/ 11 (26%)	0.305
Recurrent biliary obstruction (RBO)		32 (26%)	16 (20%)	16 (37%)	0.034
	Symptomatic stent migration	3 (3%)	3 (4%)	0	0.551
	Stent occlusion	27 (22%)	11 (14%)	16 (37%)	0.005
	Ingrowth	16 (59%)	2 (18%)	14 (88%)	<0.001
	Sludges	7 (26%)	5 (45%)	2 (13%)	0.084
	Overgrowth	4 (15%)	4 (36%)	0	0.019
	Stent removal due to AEs	2	2 (2%; PEP, cholecystitis)	0	0.543
Asymptomatic stent migration		5 (4%)	5 (6%)	0	0.139
Success rate for stent removal		100% (13/ 13)	100% (13/ 13)	0	0.004
Method of re‐intervention for RBO					
	SEMS placement	19(59%)	12 (75%)	7 (44%)	0.974
	Stent exchange	10 (31%)	10 (63%)	0	—
	Stent‐in‐stent	9 (28%)	2 (12%)	7	—
	PS placement	8 (25%)	2 (13%)	6 (38%)	0.094
	Stent exchange	2 (6%)	2 (13%)	0	—
	Stent‐in‐stent	6 (19%)	0	6 (38%)	—
	Percutaneous drainage	1 (3%)	1 (6%)	0	1.000
	Cleaning or no treatment	4 (13%)	1 (6%)	3 (18%)	0.120
Treatment‐related adverse events		17 (13%)	12 (15%)	5 (12%)	0.623
	Pancreatitis	8 (6%)	8 (10%)	0	0.050
	Cholecyctitis	7 (5%)	3 (3%)	4 (9%)	0.234
	Bleeding	2 (2%)	1 (1%)	1 (2%)	1.000
Death in the observation period		99 (80%)	65 (80%)	34 (79%)	0.876

Abbreviations: AEs: adverse events, EPBD: endoscopic papillary balloon dilation, ESBD: endoscopic sphincterotomy followed by balloon dilation, EST: endoscopic sphincterotomy, PS: plastic stents, RBO: recurrent biliary obstruction, SEMS: self‐expandable metal stents.

Regarding stent type selection, covered SEMS were used in 81 patients (65%). Three of the eight participating hospitals exclusively used covered SEMS, while the remaining five hospitals used both covered and uncovered SEMS. The choice of stent type was at the discretion of the endoscopist.

Papillary manipulation, including EST (60%), EPBD (22%), and ESBD (18%), was performed in 102 patients (82%) before stent insertion. Stents were placed across the papilla in 85 patients (68%).

RBO occurred in 32 patients (26%), comprising stent migration in three patients (2%), stent occlusion in 27 patients (22%), and stent removal due to other reasons in two patients (2%). Among these, 13 patients underwent an attempt to remove the occluded stent, and all cases (100%) were successfully removed. The median time to stent removal was 113 days (IQR: 22–176 days), with the longest duration before removal being 418 days; however, this stent was removed without difficulty.

Reintervention following RBO included SEMS replacement in 19 patients (59%), plastic stent placement in eight (25%), additional percutaneous drainage in one (3%), and stent cleaning or no treatment in four (13%).

Treatment‐related AEs were observed in 16 patients (13%), including pancreatitis in eight patients (6%), cholecystitis in six (5%), and hemorrhage in two (2%). Among the eight cases of pancreatitis, two cases (25%) required SEMS removal within a few days after ERCP. Five cases (63%) were classified as mild, while the remaining three cases (37%) were moderate, as hospitalization was prolonged by approximately one week. All eight patients recovered completely.

Cholecystitis occurred in four patients with cholangiocarcinoma, two with pancreatic head cancer, and one with gallbladder cancer. All seven cases were classified as moderate and required percutaneous transhepatic gallbladder drainage (PTGBD) or EUS‐guided gallbladder drainage (EUS‐GBD). Among these cases, cancer invasion of the cystic duct was observed in three patients. However, there was no significant difference in the incidence of cholecystitis in relation to cystic duct invasion (*p* = 0.723).

The two cases of hemorrhage were both mild and occurred in patients who were not on antithrombotic therapy. In both cases, hemostasis was successfully achieved with simple compression.

During the observation period, 99 patients (80%) died.

### 3.3 Comparison of Outcomes between Covered and Uncovered SEMS

Covered SEMS were placed in 81 patients (65%), while uncovered SEMS were used in 43 patients (35%).

When comparing patient characteristics between the two groups, cases with a naïve papilla were more frequent in the uncovered SEMS group, and the median length of biliary stricture was also longer in this group (Table 1). In terms of treatment‐related factors, 10‐mm stents were more commonly used in the covered SEMS group, whereas EST was more frequently performed in the uncovered SEMS group. However, there was no significant difference in the technical and clinical success rates between the two groups (Table 2).

The rate of RBO was significantly higher in the uncovered SEMS group than in the covered SEMS group (37% vs. 20%, *p* = 0.034), particularly in terms of stent occlusion (37% vs. 14%, *p* = 0.005). Ingrowth represented the most common cause of stent occlusion and was significantly more frequent in the uncovered stent group than in the covered stent group (*p* < 0.001). One case of RBO in the covered SEMS group was associated with a stent fracture. In this case, RBO occurred 182 days after stent placement due to tumor overgrowth, although the stent fracture itself was not the direct cause of obstruction. We performed an additional analysis by dividing patients into two groups, with and without RBO, to identify factors associated with RBO (Table S1). In the univariate analysis, the use of antithrombotic agents and the type of stent (covered vs. uncovered) were extracted as associated factors. In the multivariate analysis, the use of antithrombotic agents was no longer significant, and only the type of stent remained as an independent factor associated with RBO.

Asymptomatic stent migration due to tumor shrinkage was observed in 5 cases in which covered SEMS were placed. Stent removal was attempted only in the covered SEMS group, and all 13 stents were successfully removed (Table S2). Uncovered SEMS were not attempted to be removed.

There was no significant difference in the rate of treatment‐related AEs between the two groups. However, pancreatitis did not occur in the uncovered SEMS group and tended to be less frequent in this group compared with the covered SEMS group (10% vs. 0%, *p* = 0.050).

### 3.4 TRBO and Patient Survival Curves between Covered and Uncovered SEMS

**FIGURE 2:**
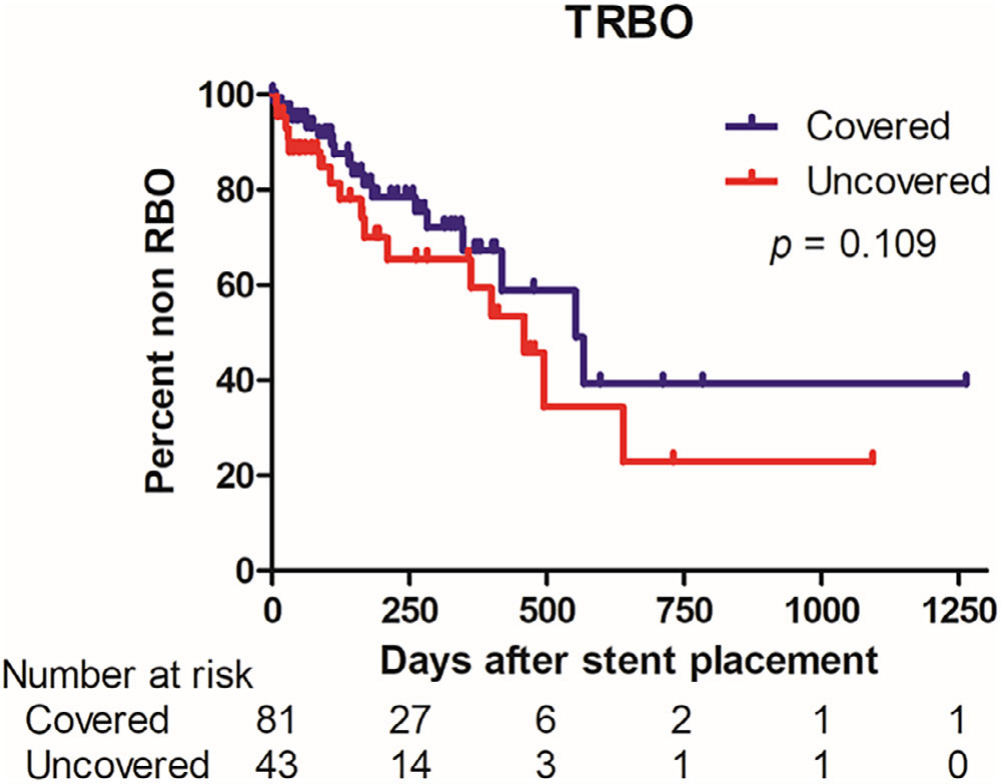
Kaplan‐Meier curves for time to recurrent biliary obstruction (TRBO) comparing covered and uncovered laser‐cut self‐expandable metal stent (SEMS). The median cumulative TRBO was 567 days (95% confidence interval, 525–608 days) for covered SEMS and 459 days (95% confidence interval, 340–577 days) for uncovered SEMS. Although the difference was not statistically significant (*p* = 0.109), the cumulative patency rate remained consistently higher for covered SEMS throughout the observation period.

**FIGURE 3:**
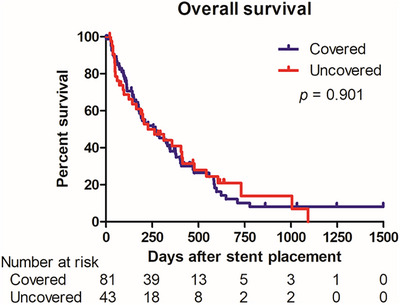
Kaplan‐Meier curves for patient survival comparing covered and uncovered laser‐cut self‐expandable metal stent (SEMS). The median cumulative patient survival was 277 days (95% confidence interval, 155–398 days) for covered SEMS and 227 days (95% confidence interval, 72–381 days) for uncovered SEMS. No significant difference was observed between the groups (*p* = 0.901), and the survival curves were nearly identical.

TRBO (Figure 2) and patient survival (Figure 3) were estimated using Kaplan‐Meier curves and compared between the covered and uncovered SEMS groups using the log‐rank test.

The median cumulative TRBO was 567 days (95% confidence interval [CI]: 525–608 days) for covered SEMS and 459 days (95% CI: 340–577 days) for uncovered SEMS. Although the difference was not statistically significant (*p* = 0.109), the cumulative non‐RBO rate remained consistently higher in the covered SEMS group throughout the observation period (Figure 2).

Similarly, the median cumulative patient survival was 277 days (95% CI: 155–398 days) for covered SEMS and 227 days (95% CI: 72–381 days) for uncovered SEMS. There was no significant difference in patient survival between the two groups (*p* = 0.901), and their survival curves were nearly identical (Figure 3).

We also examined the impact of chemotherapy (Table S3) and stent placement method (above the papilla vs. across the papilla, Table S4) on RBO rate, TRBO, and treatment‐related AEs. When analyzed separately for covered and uncovered stents, neither factor showed a significant difference.

## 4 Discussion

In this study, stent placement succeeded in all 124 patients. Although braided stents usually achieve a 97%–99% success rate [5], failures may occur from unintended movement such as shortening or jumping. The 100% success rate here likely reflects the ease of deploying laser‐cut stents, which show minimal unintended movement [7, 19].

### 4.1 Stent Migration and Removability

Stent migration occurred in eight patients (6%), all with covered stents; five were asymptomatic due to tumor shrinkage, and three caused RBO. A systematic review reported a 9.8% symptomatic migration rate for covered stents [20], whereas covered laser‐cut SEMS in this study had a lower rate (3.7%). This may be explained by their higher radial force—about twice that of braided SEMS in our prior study [4, 21]—and their pointed wire structure, which anchors the stent to surrounding tissue.

Covered stents were removed in all 13 attempted cases (Table S2). Twelve were easily retrieved with a snare, but one bile duct cancer case was difficult, suggesting removal may be more challenging in this setting. Previous reports showed variable success: Yamada et al. achieved removal in all six cases [8], while Hasegawa et al. succeeded in six of 12 [11]. Despite such variability, our results indicate that covered laser‐cut SEMS can generally be removed effectively when required.

### 4.2 Drainage Efficiency

Covered laser‐cut SEMS showed significantly lower RBO rates than uncovered stents. In multivariate analysis, stent type was the only independent factor, mainly due to fewer stent occlusions. Tumor ingrowth was significantly more frequent in uncovered stents, explaining their higher RBO incidence, consistent with prior reports on braided stents [2].

TRBO did not differ significantly between groups, likely due to the limited sample size. Based on Yamashita et al. [2], about 150 patients (75 per group) would be required to detect a significant difference, whereas our study included 124. Nonetheless, TRBO for laser‐cut SEMS was comparable to that of braided stents in previous studies [11, 22].

### 4.3 Adverse Events

The overall AE rate did not differ significantly between groups, but pancreatitis was less frequent with uncovered SEMS (*p* = 0.050). Prior studies reported similar pancreatitis rates between covered and uncovered braided SEMS [23, 24]. We also compared above‐ and across‐placements, with no significant difference (Table S4). Covering may increase axial force [4] and thereby the risk of pancreatitis, possibly more in laser‐cut SEMS. This difference may also be related to more frequent papillary balloon dilation in covered SEMS.

One covered SEMS case showed a stent fracture. Although not linked to RBO or AE here, fractures can cause bile duct injury or kinking [25], and remain rarely reported in braided SEMS, warranting caution with laser‐cut SEMS.

### 4.4 Limitations

This study has several limitations. First, its retrospective design led to unequal case distribution between covered and uncovered SEMS groups; a randomized trial is needed to confirm these results.

Second, the sample size was small, which may explain why the Kaplan–Meier curves suggested but did not show a significant TRBO difference.

Third, ERCP procedures varied across hospitals, introducing potential selection bias in stent choice and RBO management.

## 5 Conclusion

Since the X‐SUIT NIR stent is unavailable, the present results should be regarded mainly as mechanistic insights into covered versus uncovered laser‐cut SEMS, potentially informing future stent development and application. Laser‐cut SEMS showed efficacy comparable to braided SEMS for malignant distal biliary drainage, with lower RBO rates in covered than uncovered SEMS. Covered laser‐cut SEMS may also migrate less than covered braided SEMS. However, long‐term use requires caution due to possible stent fracture.

## Conflicts of Interest

Tadakazu Hisamatsu received research grants from Boston Scientific Japan. Masao Toki received lecture fees from Olympus Corporation and advisory fees from ZEON Medical Corporation. Hiroyuki Isayama received research grants from Boston Scientific Japan, Century Medical, and FUJIFILM Corporation. This study was not supported by any of these companies. Takao Itoi is the Editor‐in‐Chief of DEN Open, and Eisuke Iwasaki is an associate editor of DEN Open. The other authors declare no conflicts of interest.

## Ethics Statement

Approval of the research protocol by an Institutional Review Board: This study was approved by the ethics committee at each participating institution (H20‐0002).

## Consent

N/A.

## Clinical Trial Registration

N/A.

## Supporting information




**Table S1**: Analysis of factors associated with recurrent biliary obstruction (RBO).

**Table S2**: Details of cases with stent removal.

**Table S3**: Recurrent biliary obstruction (RBO), time to recurrent biliary obstruction (TRBO), and adverse events (AEs) by chemotherapy.

**Table S4**: Recurrent biliary obstruction (RBO), time to recurrent biliary obstruction (TRBO), and adverse events (AEs) by stent placement methods.

## References

[1] H. Isayama , Y. Komatsu , T. Tsujino , et al., “A Prospective Randomised Study of “Covered” versus “Uncovered” Diamond Stents for the Management of Distal Malignant Biliary Obstruction,” Gut 53 (2004): 729–734. 15082593 10.1136/gut.2003.018945 PMC1774024

[2] Y. Yamashita , A. Tachikawa , T. Shimokawa , et al., “Covered versus Uncovered Metal Stent for Endoscopic Drainage of a Malignant Distal Biliary Obstruction: Meta‐analysis,” Digestive Endoscopy 34 (2022): 938–951. 35114036 10.1111/den.14260

[3] M. A. Almadi , A. Barkun , and M. Martel , “Plastic vs. Self‐Expandable Metal Stents for Palliation in Malignant Biliary Obstruction: A Series of Meta‐Analyses,” American Journal of Gastroenterology 112 (2017): 260–273. 27845340 10.1038/ajg.2016.512

[4] W. Yamagata , T. Fujisawa , T. Sasaki , et al., “Evaluation of the Mechanical Properties of Current Biliary Self‐expandable Metallic Stents: Axial and Radial Force, and Axial Force Zero Border,” Clinical Endoscopy 56 (2023): 633–649. 37032114 10.5946/ce.2022.201 PMC10565432

[5] P. Loganathan , S. Chandan , B. P. Mohan , et al., “Comparable Efficacy of Laser‐Cut and Braided Self Expanding Metallic Biliary Stent: A Systematic Review and Meta‐Analysis,” Digestive Diseases and Sciences 68 (2023): 3756–3764. 37439926 10.1007/s10620-023-08017-w

[6] M. A. Almadi , A. N. Barkun , and M. Martel , “No Benefit of Covered vs Uncovered Self‐expandable Metal Stents in Patients With Malignant Distal Biliary Obstruction: A Meta‐analysis,” Clinical Gastroenterology and Hepatology 11 (2013): 27–37. e1. 23103324 10.1016/j.cgh.2012.10.019

[7] Y. Tanisaka , M. Mizuide , A. Fujita , et al., “Can the Laser‐cut Covered Self‐expandable Metallic Stent be the First Choice for Patients With Unresectable Distal Malignant Biliary Obstruction? (With video),” Journal of Hepato‐Biliary‐Pancreatic Sciences 29 (2022): 585–593. 34390208 10.1002/jhbp.1034

[8] Y. Yamada , T. Sasaki , T. Takeda , et al., “A Novel Laser‐cut Fully Covered Metal Stent With Anti‐reflux Valve in Patients With Malignant Distal Biliary Obstruction Refractory to Conventional Covered Metal Stent,” Journal of Hepato‐Biliary‐Pancreatic Sciences 28 (2021): 563–571. 33835728 10.1002/jhbp.966

[9] M. Itonaga , T. Ogura , H. Isayama , et al., “Usefulness of a Dedicated Laser‐cut Metal Stent With an Anchoring Hook and Thin Delivery System for EUS‐guided Hepaticogastrostomy in Malignant Biliary Obstruction: A Prospective Multicenter Trial (With Video),” Gastrointestinal Endoscopy 101 (2025): 970–978. 39521097 10.1016/j.gie.2024.11.005

[10] H. Isayama , K. Kawakubo , Y. Nakai , et al., “A Novel, Fully Covered Laser‐cut Nitinol Stent With Antimigration Properties for Nonresectable Distal Malignant Biliary Obstruction: A Multicenter Feasibility Study,” Gut Liver 7 (2013): 725–730. 24312715 10.5009/gnl.2013.7.6.725 PMC3848551

[11] S. Hasegawa , T. Sato , S. Shinoda , et al., “Braided‐type Stent versus Laser‐cut‐type Stent for Patients With Unresectable Distal Malignant Biliary Obstruction: A Randomized Controlled Trial,” Gastrointestinal Endoscopy 99 (2024): 739–746. e1. 38065510 10.1016/j.gie.2023.11.057

[12] S. Marui , N. Uza , H. Yamazaki , et al., “Utility of Laser‐cut Covered Self‐expandable Metal Stents for Unresectable Malignant Distal Biliary Obstruction: A Single‐center Experience,” Endoscopy 52, no. 8: 664–668: (2020). 32316040 10.1055/a-1149-1700

[13] S. Takahashi , T. Fujisawa , M. Ushio , et al., “Retrospective Evaluation of Slim Fully Covered Self‐expandable Metallic Stent for Unresectable Malignant Hilar Biliary Obstruction,” Journal of Hepato‐Biliary‐Pancreatic Sciences 30 (2023): 408–415. 35918901 10.1002/jhbp.1221

[14] K. Kitagawa , A. Mitoro , T. Ozutsumi , et al., “Laser‐cut‐type versus Braided‐type Covered Self‐expandable Metallic Stents for Distal Biliary Obstruction Caused by Pancreatic Carcinoma: A Retrospective Comparative Cohort Study,” Clinical Endoscopy 55 (2022): 434–442. 34706489 10.5946/ce.2021.161 PMC9178141

[15] S. Ishii , T. Fujisawa , M. Ushio , et al., “Evaluation of the Safety and Efficacy of Minimal Endoscopic Sphincterotomy Followed by Papillary Balloon Dilation for the Removal of Common Bile Duct Stones,” Saudi Journal of Gastroenterology 26 (2020): 344–350. 32719239 10.4103/sjg.SJG_162_20 PMC8019135

[16] S. Ishii , H. Isayama , M. Ushio , et al., “Best Procedure for the Management of Common Bile Duct Stones via the Papilla: Literature Review and Analysis of Procedural Efficacy and Safety,” Journal of Clinical Medicine 9, no. 12 (2020): 3808. 33255554 10.3390/jcm9123808 PMC7760048

[17] S. Q. Dong , T. P. Singh , Q. Zhao , et al., “Sphincterotomy plus Balloon Dilation versus Sphincterotomy Alone for Choledocholithiasis: A Meta‐analysis,” Endoscopy 51 (2019): 763–771. 30786316 10.1055/a-0848-8271

[18] H. Isayama , T. Hamada , T. Fujisawa , et al., “TOKYO Criteria 2024 for the Assessment of Clinical Outcomes of Endoscopic Biliary Drainage,” Digestive Endoscopy 36 (2024): 1195–1210. 38845085 10.1111/den.14825

[19] M. Okuno , K. Iwata , T. Mukai , et al., “The Evaluation of Bilateral Stenting Using Braided or Laser‐cut Self‐expandable Metallic Stent for Malignant Hilar Biliary Obstruction,” Surgical Endoscopy 37 (2023): 8489–8497. 37759143 10.1007/s00464-023-10457-4

[20] G. Vanella , C. Coluccio , A. Cucchetti , et al., “Fully Covered versus Partially Covered Self‐expandable Metal Stents for Palliation of Distal Malignant Biliary Obstruction: A Systematic Review and Meta‐analysis,” Gastrointestinal Endoscopy 99 (2024): 314–322. e19. 37813199 10.1016/j.gie.2023.10.023

[21] H. Isayama , Y. Nakai , Y. Toyokawa , et al., “Measurement of Radial and Axial Forces of Biliary Self‐expandable Metallic Stents,” Gastrointestinal Endoscopy 70 (2009): 37–44. 19249766 10.1016/j.gie.2008.09.032

[22] H. Kogure , S. Ryozawa , I. Maetani , et al., “A Prospective Multicenter Study of a Fully Covered Metal Stent in Patients With Distal Malignant Biliary Obstruction: WATCH‐2 Study,” Digestive Diseases and Sciences 63 (2018): 2466–2473. 29218484 10.1007/s10620-017-4875-5

[23] J. J. Telford , D. L. Carr‐Locke , T. H. Baron , et al., “A Randomized Trial Comparing Uncovered and Partially Covered Self‐expandable Metal Stents in the Palliation of Distal Malignant Biliary Obstruction,” Gastrointestinal Endoscopy 72 (2010): 907–914. 21034891 10.1016/j.gie.2010.08.021

[24] K. Kawakubo , H. Isayama , Y. Nakai , et al., “Risk Factors for Pancreatitis Following Transpapillary Self‐expandable Metal Stent Placement,” Surgical Endoscopy 26 (2012): 771–776. 22011943 10.1007/s00464-011-1950-4

[25] C. Zhou , B. Wei , J. Wang , et al., “Self‐Expanding Metallic Stent Fracture in the Treatment of Malignant Biliary Obstruction,” Gastroenterology Research and Practice 2018 (2018): 6527879. 29849597 10.1155/2018/6527879 PMC5914116

